# Supplementary material for: The casts of Pompeii: Post-depositional methodological insights
Source: PLoS One. 2023 Aug 23;18(8):e0289378. doi: 10.1371/journal.pone.0289378 (PMC10446210; doi:10.1371/journal.pone.0289378)
Supplement: S1 File — (DOCX) [file pone.0289378.s001.docx]

**S1 File. Biological profile of the casts**

**The cast #57*** (Porta Nola. State inventory no. 90454. *new chronological classification 2019 Pompeii Archaeological Park) is an adult individual (25-30 years old) of masculine sex, divided into 6 pieces: one is the main mold (skull, chest and part of the limbs), two are from the upper left limb and three are from the lower right limb. The individual is supine and has some flexed and asymmetrical anatomical areas. The skull is inclined to the right, forming a 45º angle, supporting part of the occipital and parietal on the plane. The chest is straight presenting its coxofemoral joint slightly rotated towards its medial aspect (the left more than the right). Regarding the upper limbs, the right arm is incomplete at the level of the distal epiphysis of the humerus. The fragment belonging to the forearm has not yet been found. The position of the arm is stretched out at an angle of 90 degrees to the chest of the individual. From the left arm only half humeral shaft is available, which is attached to the chest. The left leg has an antero-lateral appearance, that is, the leg is slightly rotated inward and possibly the foreleg bent backwards giving an angle greater than 90º. It should be noted that this is fractured at the knee. The remains of the proximal epiphysis of the broken tibia have been located as a result of the insertion of the irons from old restorations. Finally, the right leg has a slightly flexed position showing its antero-lateral face, with an angle of more than 90º, and limb is fragmented at the height of the first half of the femural shaft. Also, a loss of plaster was observed on its back and front.

**The cast #62*** (Porta Nola. State inventory no. 90453. *new chronological classification 2019 Pompeii Archaeological Park) is an adult individual (25-30 years old) of female sex. She has interlaced into the left hand-forearm, a rope that would go over the left shoulder to carry a sack or bag with belongings. The original position of the subject was prone (face-down) with the head resting on the face, slightly tilted to the right resting on the facial bones, mandible, frontal and left zygomatic. The right arm was together with the elbow, fully flexed and bent with the hand under the right shoulder. The left arm was with the elbow flexed to 30º, with the hand under the left hemithorax. The legs were parallel following the longitudinal axis of the body. The right leg with the knee extended and the left with the knee slightly bent.

**The cast #58*** (Porta Nola. State inventory no. 90455. *new chronological classification 2019 Pompeii Archaeological Park) is an adult individual (20-25 years old) of masculine sex. This individual was originally found supine, with the upper extremities separated from the body. It was divided into seven parts before the restoration and the following bones can be observed: frontal, parietal and left temporal; part of the left molar, zygomatic, the mandible is visible from the lower part of the left branch at the height of the mental foranem, part of the teeth of the upper jaw and mandible are also visible. It appears to preserve the morphology of the right ear. It has stone concretions (lapilli) in the facial area (maxillary and nasal), more prominent in the right than left area. There are seven ribs on the left side (4th to 10th). As for the upper limbs, the bones of the right humerus are visible (which has been moved by inserting towards the thorax with a pause at the level of the coronoid fossa).

Head facing forward but slightly inclined to the left, the trunk is straight, the right arm is apparently attached to the torso and bent from the elbow, leaving the forearm separated from the body and forming an angle of 65 ° to the arm. The left arm is raised to shoulder height, bent from the elbow towards the individual's face, forming an angle of approximately 90°. It appears in a sitting position, with its left leg bent at the knee at a 90°angle. The right leg is semi-flexed and turned clockwise, leaving the medial part exposed. The following bone fragments associated with the individual can be observed: The right forearm from the elbow joint to the proximal phalanges; the section of the epiphysis of the distal humerus, as well as the articular facets of the proximal distal phalanges, the left arm, from the humerus shaft and the distal epiphysis of the metacarpus and the fragment of one of the proximal epiphyses of the phalanges.

Some carpals (such as the navicular and lunate), the proximal epiphysis of the metacarpus, the proximal epiphysis of the second phalanx fragment are also visible. There is a fracture of the area beyond the shaft of the humerus that is partially in the mold of the individual. In the same skeletal section, there is a fragment of a rib and the anterior is part of the distal epiphysis of the radius, and in its side the flexed elbow joint (humerus, ulna and radius) is perfectly visible. These three bones are visible in the following way: cubitus from the extreme proximal epiphysis up to half of the diaphysis; the radius from the end of the proximal epiphysis to the first third of the diaphysis, and the distal epiphysis of the left humerus.

The leg from the proximal epiphysis / extremity of the tibia and fibula to the heel, calcaneus bone, cuboid and third and second cuneiform with the left lower limb of the mould are still visible. Parallel to the fibula runs a root that has left marks on the bone, of a reddish hue. In this area there are stone concretions that involve the legs.

The right foot has preserved its morphology in the mould; part of the trochlea of the talus and part of the bone of the metacarpus and proximal phalanges.

**The cast #64*** () (Porta Nola. State inventory no. 90461. *new chronological classification 2019 Pompeii Archaeological Park). It was identified as an adult individual between 45 and 50 years old, probably male, very altered and remodelled in an old restoration, especially in the zygomatic-facial area. The ends of both legs are missing from the knee joint. In the right knee, a chronic and prolonged arthritic lesion stands out. The individual originally arranged supine, with the upper extremities separated from the body. The right arm with the elbow is slightly flexed and the left with the elbow joint flexed 90º.

**The cast #54*** (Porta Nola. State inventory no. 90452. *new chronological classification 2019 Pompeii Archaeological Park) is an individual exhibited at the Pompeii amphitheatre exhibition. It could be estimated that it is a female adult (35-40 years) determined by the macroscopic observation of the cranial, parietal and occipital bones and lambdoid and sagittal sutures. The individual was found on the left lateral decubitus, with the head resting on the left temporal parietal and the right arm with the elbow fully flexed and the forearm next to the chest. The left arm flexed 90º with the forearm next to the abdomen is in a position to hold a cane, branch, or crook. The right leg is with the knee bent 90º on the left leg while the left leg is slightly bent.

**The cast #55*** (Porta Nola. State inventory no. 90462. *new chronological classification 2019 Pompeii Archaeological Park) was identified as an adult male individual between 25 and 30 years old, the right arm showing a perimortem fracture of the distal third of the shaft of the humerus where a thick tree branch appears. This individual was originally arranged supine, with the upper extremities separated from the body, with the forearms and hands on the head. Both arms appear symmetrical and parallel with the elbows flexed 30º. The legs are separated, symmetrical and parallel, following the longitudinal axis of the body with both knees bent 45º.

**The cast #34*** (Terme Suburbane. State inventory no. 90459. *new chronological classification 2019 Pompeii Archaeological Park) is a probable male adult individual (20-25 years), identified by dimensions of the right femur. The original position of the subject was right lateral decubitus, with the head resting on the right zygomatic, parietal and temporal bones. The right arm was with the elbow flexed, separated from the body. The legs were parallel following the longitudinal axis of the body, both legs with the knees bent at 90º.
