# Supplementary material for: The casts of Pompeii: Post-depositional methodological insights
Source: PLoS One. 2023 Aug 23;18(8):e0289378. doi: 10.1371/journal.pone.0289378 (PMC10446210; doi:10.1371/journal.pone.0289378)
Supplement: S3 File — (DOCX) [file pone.0289378.s003.docx]

**S3 File. Other major and minor element graphs**

**
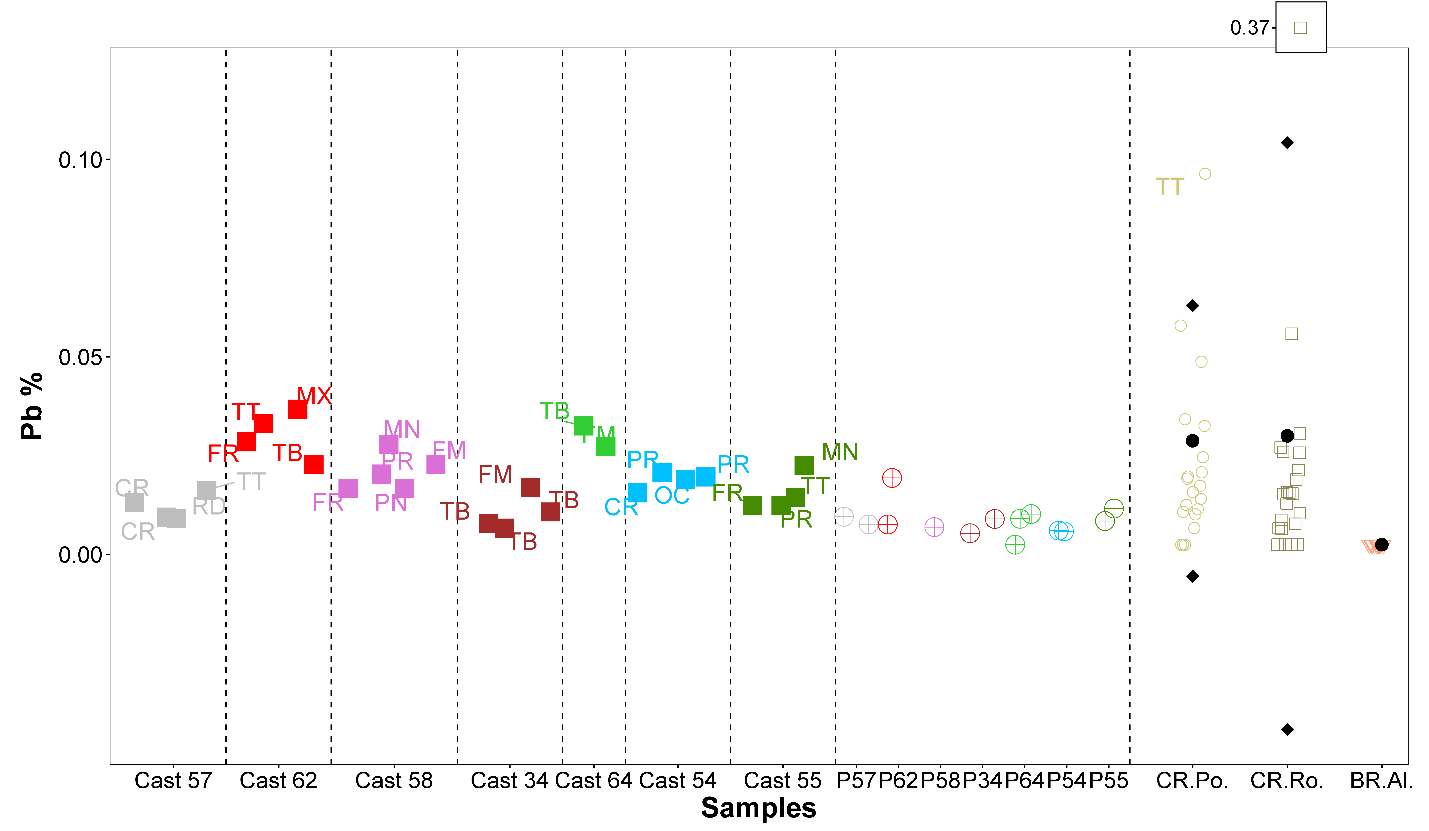
**

**Fig S1 – Pb concentrations.** Pb concentrations for all the analyzed materials. Cast, plaster (P) cremated bones from Pompeii (CR. Po.) and Rome (CR. Ro.) and buried bones from the Islamic necropolis of Vall d’ Albaida (BR. Al.). In the graph for each cast the kind of bone is indicated: cranium (CR), parietal (PR), occipital (OC), frontal (FR), maxilla (MX), mandibular (MD), tooth (TT), radius (RD), peroneal bone (PR), tibia (TB), femur (FM). •: mean; ♦: 1 standard deviation.

**
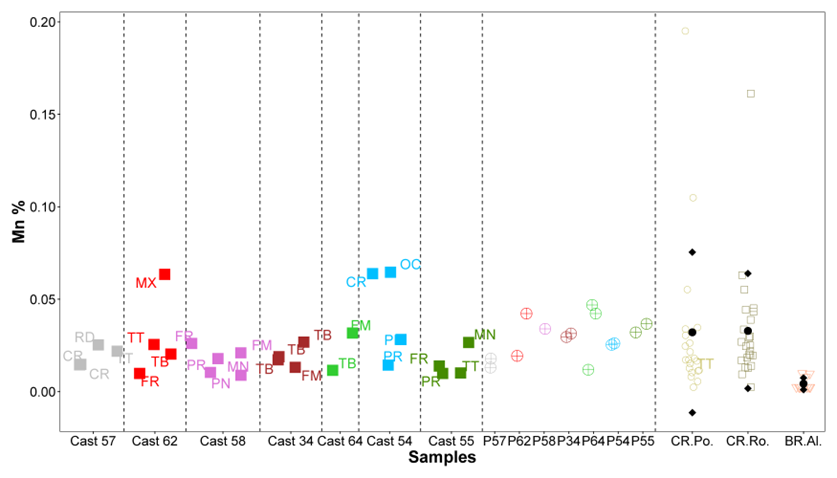
**

**Fig S2 – Mn concentrations.** Mn concentrations for all the analyzed materials. Cast, plaster (P) cremated bones from Pompeii (CR. Po.) and Rome (CR. Ro.) and buried bones from the Islamic necropolis of Vall d’ Albaida (BR. Al.). In the graph for each cast the kind of bone is indicated: cranium (CR), parietal (PR), occipital (OC), frontal (FR), maxilla (MX), mandibular (MD), tooth (TT), radius (RD), peroneal bone (PR), tibia (TB), femur (FM). •: mean; ♦: one standard deviation.

**
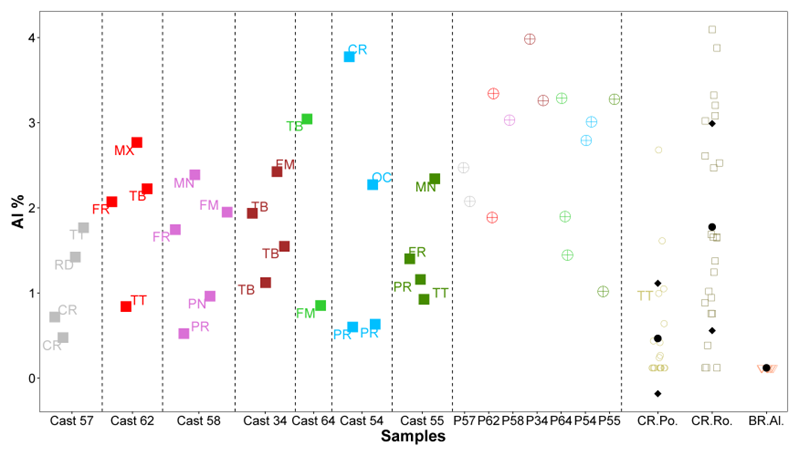
**

**Fig S3 – Al concentrations.** Al concentrations for all the analyzed materials. Cast, plaster (P) cremated bones from Pompeii (CR. Po.) and Rome (CR. Ro.) and buried bones from the Islamic necropolis of Vall d’ Albaida (BR. Al.). In the graph for each cast the kind of bone is indicated: cranium (CR), parietal (PR), occipital (OC), frontal (FR), maxilla (MX), mandibular (MD), tooth (TT), radius (RD), peroneal bone (PR), tibia (TB), femur (FM). •: mean; ♦: one standard deviation.


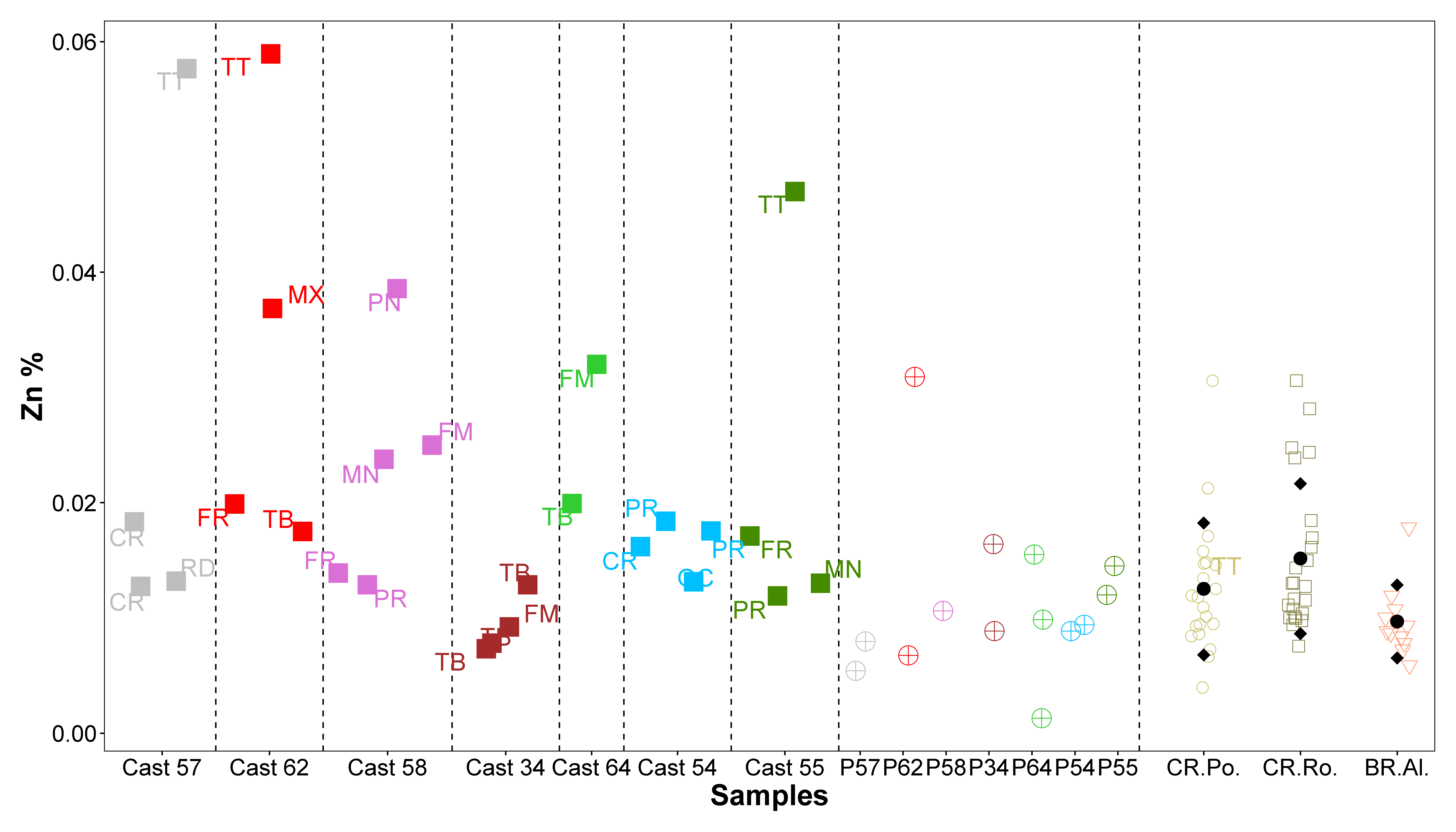


**Fig S4 – Zn concentrations.** Zn concentrations for all the analyzed materials. Cast, plaster (P) cremated bones from Pompeii (CR. Po.) and Rome (CR. Ro.) and buried bones from the Islamic necropolis of Vall d’ Albaida (BR. Al.). In the graph for each cast the kind of bone is indicated: cranium (CR), parietal (PR), occipital (OC), frontal (FR), maxilla (MX), mandibular (MD), tooth (TT), radius (RD), peroneal bone (PR), tibia (TB), femur (FM). •: mean; ♦: one standard deviation.

**
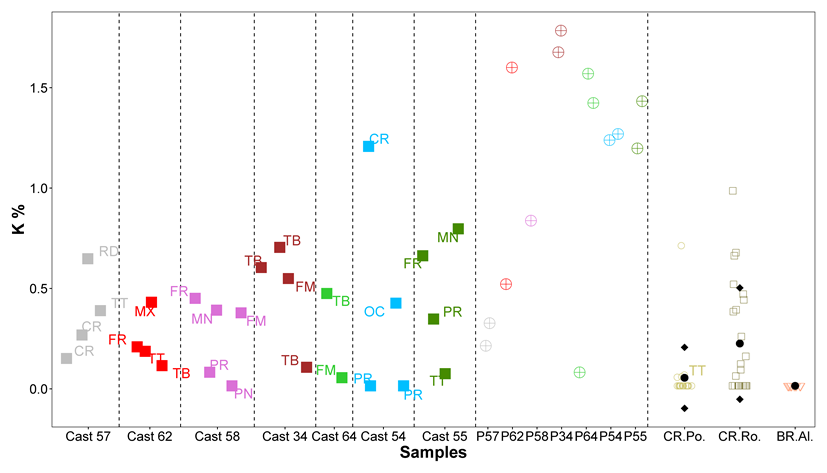
**

**Fig S5 – K concentrations.** K concentrations for all the analyzed materials. Cast, plaster (P) cremated bones from Pompeii (CR. Po.) and Rome (CR. Ro.) and buried bones from the Islamic necropolis of Vall d’ Albaida (BR. Al.). In the graph for each cast the kind of bone is indicated: cranium (CR), parietal (PR), occipital (OC), frontal (FR), maxilla (MX), mandibular (MD), tooth (TT), radius (RD), peroneal bone (PR), tibia (TB), femur (FM). •: mean; ♦: one standard deviation.

**
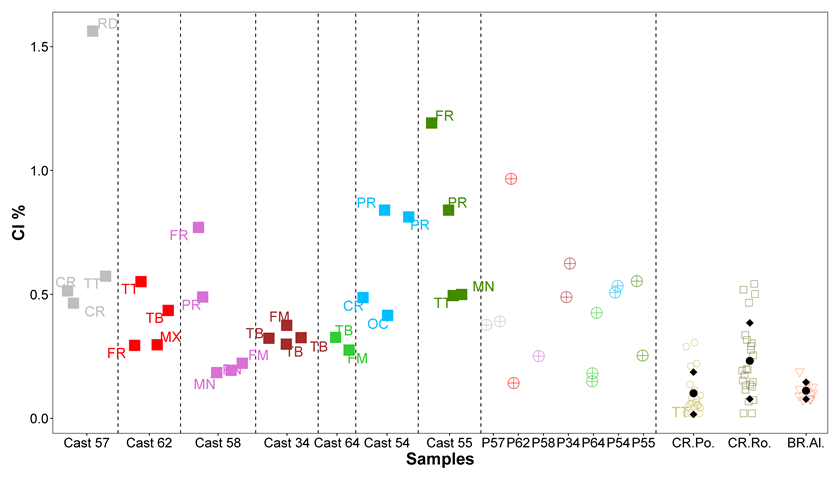
**

**Fig S6 – Cl concentrations.** Cl concentrations for all the analyzed materials. Cast, plaster (P) cremated bones from Pompeii (CR. Po.) and Rome (CR. Ro.) and buried bones from the Islamic necropolis of Vall d’ Albaida (BR. Al.). In the graph for each cast the kind of bone is indicated: cranium (CR), parietal (PR), occipital (OC), frontal (FR), maxilla (MX), mandibular (MD), tooth (TT), radius (RD), peroneal bone (PR), tibia (TB), femur (FM). •: mean; ♦: one standard deviation.

**
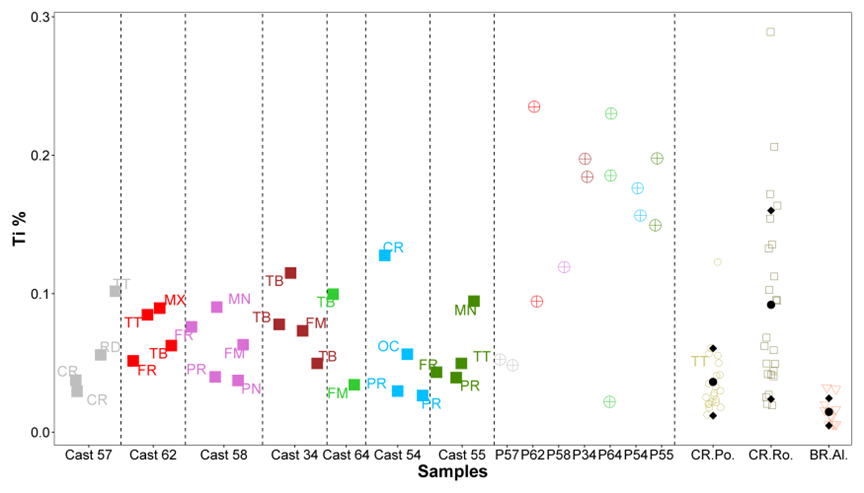
**

**Fig S7 – Ti concentrations.** Ti concentrations for all the analyzed materials. Cast, plaster (P) cremated bones from Pompeii (CR. Po.) and Rome (CR. Ro.) and buried bones from the Islamic necropolis of Vall d’ Albaida (BR. Al.). In the graph for each cast the kind of bone is indicated: cranium (CR), parietal (PR), occipital (OC), frontal (FR), maxilla (MX), mandibular (MD), tooth (TT), radius (RD), peroneal bone (PR), tibia (TB), femur (FM). •: mean; ♦: one standard deviation.

**
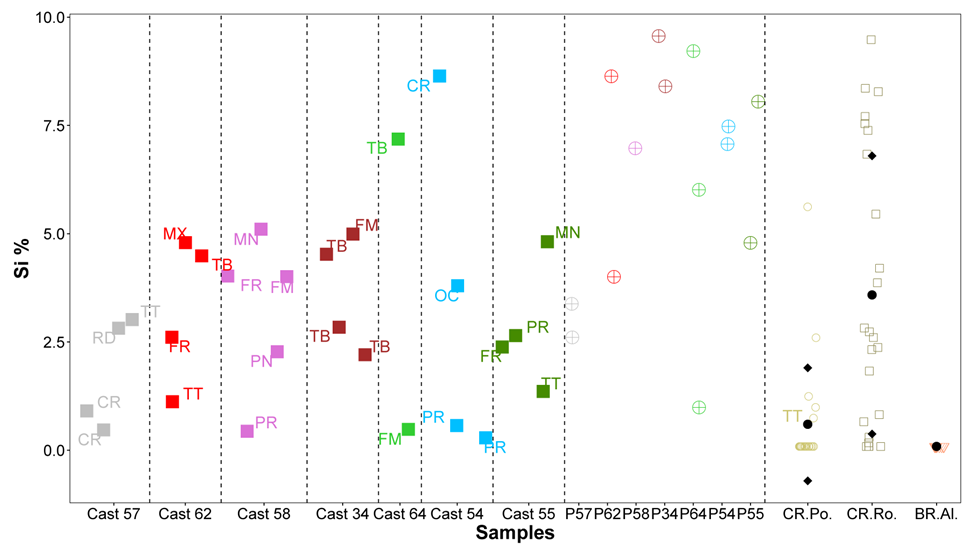
**

**Fig S8 – Si concentrations.** Si concentrations for all the analyzed materials. Cast, plaster (P) cremated bones from Pompeii (CR. Po.) and Rome (CR. Ro.) and buried bones from the Islamic necropolis of Vall d’ Albaida (BR. Al.). In the graph for each cast the kind of bone is indicated: cranium (CR), parietal (PR), occipital (OC), frontal (FR), maxilla (MX), mandibular (MD), tooth (TT), radius (RD), peroneal bone (PR), tibia (TB), femur (FM). •: mean; ♦: one standard deviation.

**
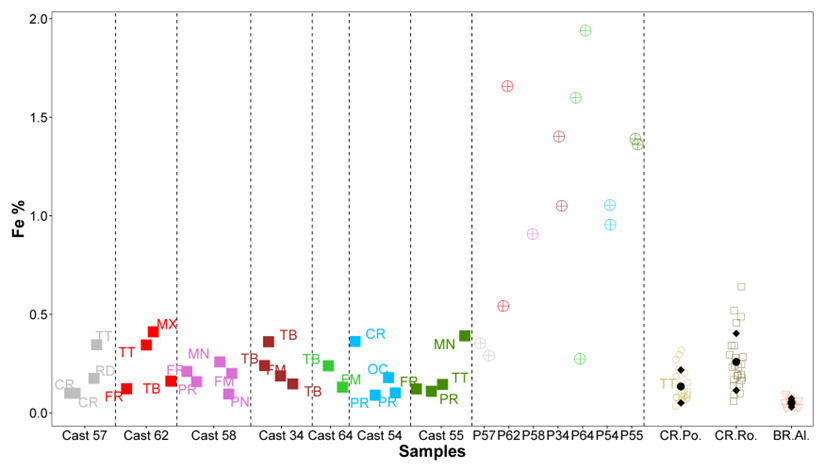
**

**Fig S9 – Fe concentrations.** Fe concentrations for all the analyzed materials. Cast, plaster (P) cremated bones from Pompeii (CR. Po.) and Rome (CR. Ro.) and buried bones from the Islamic necropolis of Vall d’ Albaida (BR. Al.). In the graph for each cast the kind of bone is indicated: cranium (CR), parietal (PR), occipital (OC), frontal (FR), maxilla (MX), mandibular (MD), tooth (TT), radius (RD), peroneal bone (PR), tibia (TB), femur (FM). •: mean; ♦: one standard deviation.

**
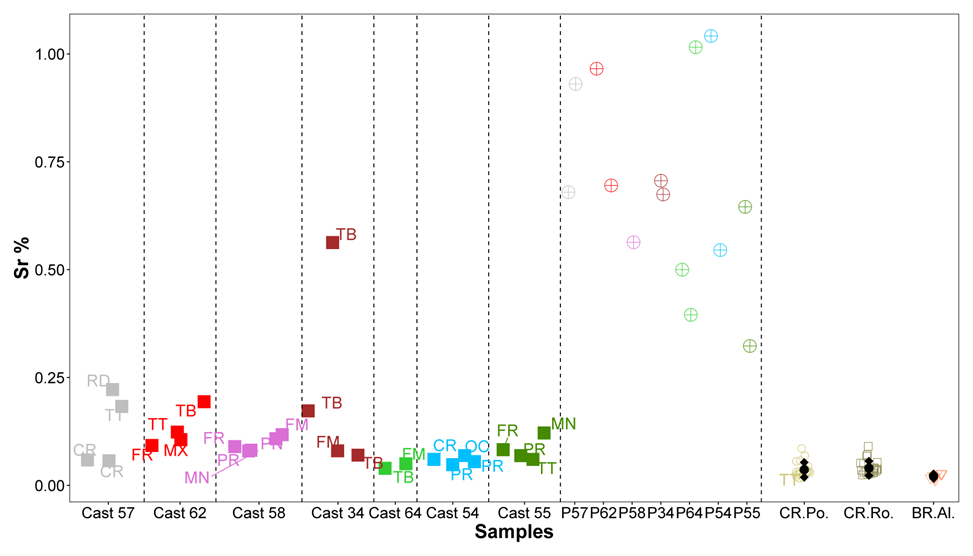
**

**Fig S10 – Sr concentrations.** Sr concentrations for all the analyzed materials. Cast, plaster (P) cremated bones from Pompeii (CR. Po.) and Rome (CR. Ro.) and buried bones from the Islamic necropolis of Vall d’ Albaida (BR. Al.). In the graph for each cast the kind of bone is indicated: cranium (CR), parietal (PR), occipital (OC), frontal (FR), maxilla (MX), mandibular (MD), tooth (TT), radius (RD), peroneal bone (PR), tibia (TB), femur (FM). •: mean; ♦: one standard deviation.

**
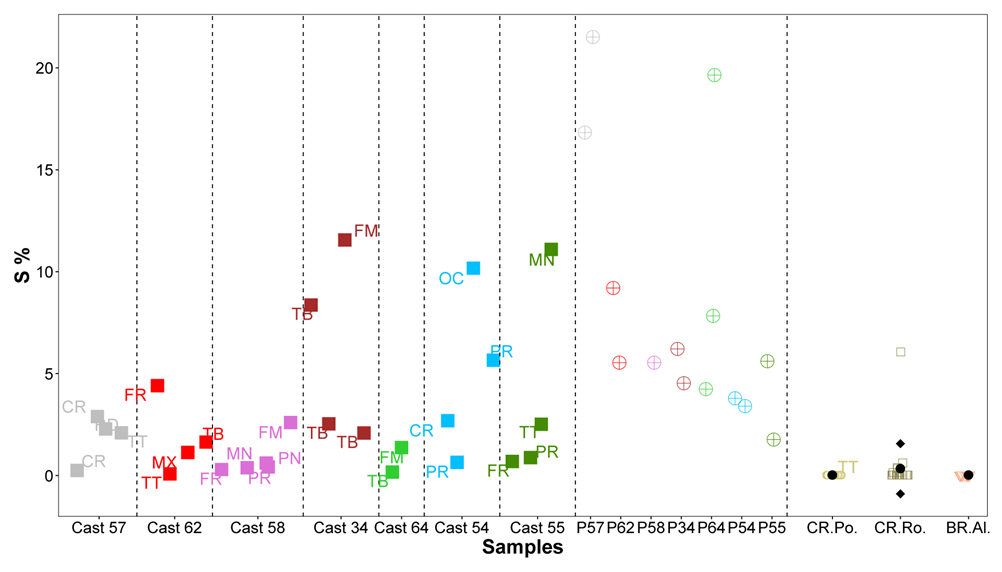
**

**Fig S11 – S concentrations.** S concentrations for all the analyzed materials. Cast, plaster (P) cremated bones from Pompeii (CR. Po.) and Rome (CR. Ro.) and buried bones from the Islamic necropolis of Vall d’Albaida (BR. Al.). In the graph for each cast the kind of bone is indicated: cranium (CR), parietal (PR), occipital (OC), frontal (FR), maxilla (MX), mandibular (MD), tooth (TT), radius (RD), peroneal bone (PR), tibia (TB), femur (FM). •: mean; ♦: one standard deviation.
