# Supplementary material for: The casts of Pompeii: Post-depositional methodological insights
Source: PLoS One. 2023 Aug 23;18(8):e0289378. doi: 10.1371/journal.pone.0289378 (PMC10446210; doi:10.1371/journal.pone.0289378)
Supplement: Attachment — Submitted filename: PONE__RESPONSE_TO_REVIEWERS.docx [file pone.0289378.s006.docx]

PONE-D-23-12449

The Casts of Pompeii: New insights

PLOS ONE

Dear Dr. Gallello,

Thank you for submitting your manuscript to PLOS ONE. After careful consideration, we feel that it has merit but does not fully meet PLOS ONE’s publication criteria as it currently stands. Therefore, we invite you to submit a revised version of the manuscript that addresses the points raised during the review process.

==============================
ACADEMIC EDITOR:

Dear Dr. Gallello,

We appreciate you submitting your manuscript to PLOS ONE and thank you for giving us the opportunity to consider your work.

I have completed my evaluation of your manuscript, which has been reviewed by four highly qualified reviewers all of whom agree it is worth to be published in PLOS ONE. Nevertheless, they have suggested some changes that will help to improve the paper.

Therefore, I invite you to resubmit your manuscript after addressing the reviewers’ comments below. When revising your manuscript, please consider all issues mentioned in the reviewers' comments carefully: please, outline every change made in response to their comments and provide suitable rebuttals for any comments not addressed. Please, note that your revised submission may need to be re-reviewed.

On the other hand, the journal staff notified me that "Manuscripts reporting paleontology and archaeology research must adhere to our policies described at [http://journals.plos.org/plosone/s/submission-guidelines#loc-paleontology-and-archaeology-research](https://urldefense.com/v3/__http:/journals.plos.org/plosone/s/submission-guidelines*loc-paleontology-and-archaeology-research__;Iw!!D9dNQwwGXtA!UAP-2qDcHRKGLmXd9qQvIwK_V6RwxAQcIA12Msm_MgqMwOM1L47NCm5QxCXgoseR-QPMOdw8O7QAnwIdQy8V$). Specifically, appropriate identification numbers for the human remains, specimens and/or samples should be provided, and the data used in the study should be publicly deposited or made accessible for replication of the study. If applicable, please ensure permission to conduct destructive sampling was obtained.”

Based on the above, in your manuscript, please provide additional information regarding the specimens used in your study. Ensure that you have reported specimen numbers and complete repository information, including museum name and geographic location.
If permits were required, please ensure that you have provided details for all permits that were obtained, including the full name of the issuing authority, and add the following statement:
'All necessary permits were obtained for the described study, which complied with all relevant regulations.'
If no permits were required, please include the following statement:
'No permits were required for the described study, which complied with all relevant regulations.'
For more information on PLOS ONE's requirements for paleontology and archaeology research, see [https://journals.plos.org/plosone/s/submission-guidelines#loc-paleontology-and-archaeology-research](https://urldefense.com/v3/__https:/journals.plos.org/plosone/s/submission-guidelines*loc-paleontology-and-archaeology-research__;Iw!!D9dNQwwGXtA!UAP-2qDcHRKGLmXd9qQvIwK_V6RwxAQcIA12Msm_MgqMwOM1L47NCm5QxCXgoseR-QPMOdw8O7QAny6MuPiQ$).

PLOS ONE values your contribution and I look forward to receiving your revised manuscript.

Yours sincerely,

Dr. Olga Spekker
==============================

Please submit your revised manuscript by Jun 30 2023 11:59PM. If you will need more time than this to complete your revisions, please reply to this message or contact the journal office at [plosone@plos.org](mailto:plosone@plos.org). When you're ready to submit your revision, log on to [https://www.editorialmanager.com/pone/](https://urldefense.com/v3/__https:/www.editorialmanager.com/pone/__;!!D9dNQwwGXtA!UAP-2qDcHRKGLmXd9qQvIwK_V6RwxAQcIA12Msm_MgqMwOM1L47NCm5QxCXgoseR-QPMOdw8O7QAnwUGlsxH$) and select the 'Submissions Needing Revision' folder to locate your manuscript file.

Please include the following items when submitting your revised manuscript:

- A rebuttal letter that responds to each point raised by the academic editor and reviewer(s). You should upload this letter as a separate file labeled 'Response to Reviewers'.
- A marked-up copy of your manuscript that highlights changes made to the original version. You should upload this as a separate file labeled 'Revised Manuscript with Track Changes'.
- An unmarked version of your revised paper without tracked changes. You should upload this as a separate file labeled 'Manuscript'.

If you would like to make changes to your financial disclosure, please include your updated statement in your cover letter. Guidelines for resubmitting your figure files are available below the reviewer comments at the end of this letter.

If applicable, we recommend that you deposit your laboratory protocols in protocols.io to enhance the reproducibility of your results. Protocols.io assigns your protocol its own identifier (DOI) so that it can be cited independently in the future. For instructions see: [https://journals.plos.org/plosone/s/submission-guidelines#loc-laboratory-protocols](https://urldefense.com/v3/__https:/journals.plos.org/plosone/s/submission-guidelines*loc-laboratory-protocols__;Iw!!D9dNQwwGXtA!UAP-2qDcHRKGLmXd9qQvIwK_V6RwxAQcIA12Msm_MgqMwOM1L47NCm5QxCXgoseR-QPMOdw8O7QAnyQSjlOc$). Additionally, PLOS ONE offers an option for publishing peer-reviewed Lab Protocol articles, which describe protocols hosted on protocols.io. Read more information on sharing protocols at [https://plos.org/protocols?utm_medium=editorial-email&utm_source=authorletters&utm_campaign=protocols](https://urldefense.com/v3/__https:/plos.org/protocols?utm_medium=editorial-email&utm_source=authorletters&utm_campaign=protocols__;!!D9dNQwwGXtA!UAP-2qDcHRKGLmXd9qQvIwK_V6RwxAQcIA12Msm_MgqMwOM1L47NCm5QxCXgoseR-QPMOdw8O7QAn5Uwhj5F$).

We look forward to receiving your revised manuscript.

Kind regards,

Olga Spekker, Ph.D.
Academic Editor
PLOS ONE

Journal Requirements:

When submitting your revision, we need you to address these additional requirements.

1. Please ensure that your manuscript meets PLOS ONE's style requirements, including those for file naming. The PLOS ONE style templates can be found at [https://journals.plos.org/plosone/s/file?id=wjVg/PLOSOne_formatting_sample_main_body.pdf](https://urldefense.com/v3/__https:/journals.plos.org/plosone/s/file?id=wjVg*PLOSOne_formatting_sample_main_body.pdf__;Lw!!D9dNQwwGXtA!UAP-2qDcHRKGLmXd9qQvIwK_V6RwxAQcIA12Msm_MgqMwOM1L47NCm5QxCXgoseR-QPMOdw8O7QAn9AqxMID$) and 
[https://journals.plos.org/plosone/s/file?id=ba62/PLOSOne_formatting_sample_title_authors_affiliations.pdf](https://urldefense.com/v3/__https:/journals.plos.org/plosone/s/file?id=ba62*PLOSOne_formatting_sample_title_authors_affiliations.pdf__;Lw!!D9dNQwwGXtA!UAP-2qDcHRKGLmXd9qQvIwK_V6RwxAQcIA12Msm_MgqMwOM1L47NCm5QxCXgoseR-QPMOdw8O7QAnyMJm3uI$)

2. In your manuscript, please provide additional information regarding the specimens used in your study. Ensure that you have reported human remain specimen numbers and complete repository information, including museum name and geographic location.

If permits were required, please ensure that you have provided details for all permits that were obtained, including the full name of the issuing authority, and add the following statement:

'All necessary permits were obtained for the described study, which complied with all relevant regulations.'

If no permits were required, please include the following statement:

'No permits were required for the described study, which complied with all relevant regulations.'

For more information on PLOS ONE's requirements for paleontology and archeology research, see [https://journals.plos.org/plosone/s/submission-guidelines#loc-paleontology-and-archaeology-research](https://urldefense.com/v3/__https:/journals.plos.org/plosone/s/submission-guidelines*loc-paleontology-and-archaeology-research__;Iw!!D9dNQwwGXtA!UAP-2qDcHRKGLmXd9qQvIwK_V6RwxAQcIA12Msm_MgqMwOM1L47NCm5QxCXgoseR-QPMOdw8O7QAny6MuPiQ$)

[Note: HTML markup is below. Please do not edit.]

Reviewers' comments:

Reviewer's Responses to Questions

**Dear editor,**

**We are really grateful for giving us the possibility to revise the manuscript and answer the reviewer’s comments. We have worked hard to address all the points raised by the four referees and these have consistently improved the quality of our manuscript. Therefore, we really thank the reviewers for their work.**

**Reviewer #1 and Reviewer #4 comments and suggestions in the corresponding files together with the Reviewers #2 and #3 have been taken into account and reported in the new version of the manuscript in revision mode with our answers and comments.**

**Comments to the Author**

1. Is the manuscript technically sound, and do the data support the conclusions?

The manuscript must describe a technically sound piece of scientific research with data that supports the conclusions. Experiments must have been conducted rigorously, with appropriate controls, replication, and sample sizes. The conclusions must be drawn appropriately based on the data presented.

Reviewer #1: Partly

Reviewer #2: Yes

Reviewer #3: Partly

Reviewer #4: Yes

2. Has the statistical analysis been performed appropriately and rigorously?

Reviewer #1: Yes

Reviewer #2: Yes

Reviewer #3: Yes

Reviewer #4: Yes

3. Have the authors made all data underlying the findings in their manuscript fully available?

The [PLOS Data policy](https://urldefense.com/v3/__http:/www.plosone.org/static/policies.action*sharing__;Iw!!D9dNQwwGXtA!UAP-2qDcHRKGLmXd9qQvIwK_V6RwxAQcIA12Msm_MgqMwOM1L47NCm5QxCXgoseR-QPMOdw8O7QAn6MSPtfR$) requires authors to make all data underlying the findings described in their manuscript fully available without restriction, with rare exception (please refer to the Data Availability Statement in the manuscript PDF file). The data should be provided as part of the manuscript or its supporting information, or deposited to a public repository. For example, in addition to summary statistics, the data points behind means, medians and variance measures should be available. If there are restrictions on publicly sharing data—e.g. participant privacy or use of data from a third party—those must be specified.

Reviewer #1: Yes

Reviewer #2: Yes

Reviewer #3: Yes

Reviewer #4: Yes

4. Is the manuscript presented in an intelligible fashion and written in standard English?

PLOS ONE does not copyedit accepted manuscripts, so the language in submitted articles must be clear, correct, and unambiguous. Any typographical or grammatical errors should be corrected at revision, so please note any specific errors here.

Reviewer #1: Yes

Reviewer #2: Yes

Reviewer #3: Yes

Reviewer #4: No

5. Review Comments to the Author

Please use the space provided to explain your answers to the questions above. You may also include additional comments for the author, including concerns about dual publication, research ethics, or publication ethics. (Please upload your review as an attachment if it exceeds 20,000 characters)

Reviewer #1: This review follows the thematic points provided by Plos One.

1. The study presents the results of original research.
Yes

2. Results reported have not been published elsewhere.
Correct - they have not

3. Experiments, statistics, and other analyses are performed to a high technical standard and are described in sufficient detail.

Partial. Bioarchaeological analysis – detail on what methods used for sex/age are needed. Currently it mostly states the sex, age result, and describes body position. If the profiling is obtained from other researchers, this needs citation.
pXRF - some clarity on method needed (see final paragraph below).

**We are grateful to the Reviewer #1 for the revision of our manuscript. The physical anthropological analysis in the casts was carried out directly in the field by one of the authors (Dr Llorence Alapont). The methodology employed to determine sex, age and all the anthropological aspects for the study of the 7 casts and the other reference collections follows the standardized parameters implemented by other authors. In the revised version of the manuscript, in methods section a new paragraph “Anthropological study” has been added and citations included as requested by Reviewer #1. About pXRF analysis the methodological aspects are clarified in the corresponding paragraph pXRF.**

4. Conclusions are presented in an appropriate fashion and are supported by the data.
Yes – but more can be brought out on the elemental analysis

**Thanks, in conclusion sections some lines have been added to enhance the elemental results.**

5. The article is presented in an intelligible fashion and is written in standard English.
Yes – I have added comments on some bits for clarification but it’s quite nit-picky – it’s well written.

**Thanks, in the new version of the manuscript, we have taken into consideration the language revision.**

6. The research meets all applicable standards for the ethics of experimentation and research integrity.
This research investigates a moment of trauma and death among the human population in Pompeii. It directly handles the material in question. Ethics statement said NA but I feel this requires some ethics statement.

**Thanks. In ethics section “Ethics statement” we have added that no samples have been collected from the casts and direct non-destructive analysis were carried.**

**In all the case including burned Pompeii bones from pre Vesuvius eruption and bones from Islamic population the permit have been regularly obtained following the Spanish, Italian, European and International rules and regulations.**

7. The article adheres to appropriate reporting guidelines and community standards for data availability.
Yes – but the Cu data is missing from supplementary information.

**Thanks for your comment, copper data were not shown because they were below the limit of detection for all the analysed materials and remains. Therefore, Cu has been removed from the manuscript revised version.**


This was an interesting article to read, thank you for sending it for review. It was concise and to the point, and presented some effective images and reading.
I have added detailed comments to the manuscript to read through – it’s mostly on some bits of clarity and should be quite quick to address.

**Thanks for the Reviewer #1 positive comments. We believe that this study is pushing forward to develop a non-destructive methodological approach for extraordinary remains like the casts of Pompeii. Consequently, we had the possibility to determine the chemical profiles of bones and plaster of some the casts for the first time, cross-referencing the data with the anthropological research. This allowed us to better understand perimortem and post-mortem processes.**


However, I can’t ascertain what the impact of the pXRF work was. The research is framed around the bioarchaeological and elemental analysis bringing new insight but the bioarch work is quite routine on the Pompeii casts, and the results/discussion have limited content on what exactly the elemental analysis showed – what interpretation have you gathered from the data? What I can mostly see is that plaster and bone are different elemental compositions – which is expected as they’re entirely different materials. This will need strengthening and more depth in discussion to really bring the value out of your large dataset.

**Thanks for the Reviewer #1 comments that give us the opportunity to clarify the role of the chemical information obtained by pXRF analysis in inhumated burned remains and plaster. Once obtained the results the first steps were to develop an approach to verify if the plaster is affecting the chemical compounds of the bones. To do it, we statistically compare the elemental contents of inhumated bones burned bones and casts bones with casts plaster to select the cast bones minimally affected by the plaster contamination. The next step was to compare inhumated bones with Pompeii pre Vesuvian and Roma Ostiense burned bones collections, where we could observe chemical differences between these two big groups, that we explain with the thermal impact in the structure of the bones that causes the loss of some major elements during post-depositional processes, as confirmed by previous studies**. **As mentioned, “probably the thermal impact together with post-depositional processes induced the leaching of carbonates and phosphates and this could explain the lower calcium and phosphorous concentrations in burned bones [25, 26]”. Once we had made sure that these observations were “true” by running statistical analysis of the obtained chemical information, we could compare the Casts bones with the inhumated and burned bones, observing that the composition of the stated casts was similar to those of the burned bones. That gives us an important input to develop hypothesis about the post-mortem exposure of the Pompeii remains to quite high temperature being their death caused by the asphyxia as showed by anthropological studies and the preservation of garments.**

**Following the Reviewer #1 comments a paragraph** “**The elemental data” has been added** **in the discussion section to further enhance the important information provided by pXRF analyses.**

The pXRF method also needs some clarity as it currently poses a little risk. Did you scan samples once, in triplicate, or other? If once, this needs to be repeated, bones are highly variable. Did you achieve infinite thickness in the scans? How did you scan the bones, was it in direct contact with the cleaned bone surface or was it through the plaster? pXRF and XRF beams don’t penetrate that far into the material and will be interfered by the plaster.

**Thanks for the comments, all the materials and remains were measured in triplicate in the same point and the new version of the manuscript this is now specified and in Supplementary Information (S2 File) the standard deviation has been added to observe the measurements variability. For all the seven studied casts, the measurements were carried out in the exposed bones, not in the bones inside the plaster. The casts presented with some bone remains out of the plaster although partially fixed in the plaster surface. The exposed parts of the bones were carefully cleaned in their surface to remove the dust. Once cleaned the analytical zone of the pXRF was pressed against the bone surface and measured. In the same way the plaster surface was measured** **to control bones contamination. To make sure that the infinite thickness is met just the most compact and thickest remains and plaster parts were measured. Furthermore some previous tests in similar bones and plaster employing pXRF were carried out in our labs under controlled conditions to evaluate the reliability and strength of the analytical method.**

**All the Reviewer #1 comments reported in the manuscript revised mode version are answered as comments in the attached document.**

Reviewer #2: The paper deals with the detail analysis of casts at Pompei with the aim of understanding the cause of death of people being impacted by the last phases of the Vesuvius eruption of 79 AD. The research is based mostly on bioarcheological data consisting of chemical measurements, by portable XRF, on bones and casts. Interpretation, namely asphyxia, is coherent with recent papers assessing the fate of people dying after the eruption based on volcanological interpretation. This multidisciplinary convergence enforces the interpretation of the natural event, which is useful also for assessing the hazard potential of devastating eruptions of Vesuvius. For these reasons I believe the paper deserves to be published on a high impact journal as PLOSONE.

**We are really grateful to Reviewer #2 for his positive comments. Therefore this study shows a multidisciplinary approach cross-referencing pXRF chemical data with anthropological research and previous published research, supporting a death for asphyxia of the studied individuals and adding information about perimortem and post-mortem processes. Finally we developed a non-destructive methodological approach for evaluating plaster contamination to discard plaster contaminated bones and avoid misleading interpretations about bone casts original chemical contents.**

Moderate revision is recommended, after consideration of the following main points

1) English needs a careful revision especially of the casts description

**Thanks, in the new version of the manuscript the language has been revised, especially in the Results section “Bioarchaeological analysis on casts”.**

2) People escaping from Pompei after the first phase of the eruption were invested by the dilute pyroclastic density currents of the final phase which were caused by the eruptive column collapse. The passage of these currents lasted for several minutes and even though the temperature of the gas-ash mixture was not very high it cannot be breath for long, causing asphyxia. Therefore, asphyxia was caused by the passage of the pyroclastic density current itself, not by ash being suspended as resulting from the previous fallout of pumice of the first phase of the eruption. Afterward, the deposition from the current formed a layer of hot ash that had thermal impact on corpse of people that have already died for asphyxia and were lying on the ground. I do suggest authors to revise the paper in the discussion section as be more consistent with such a reconstruction of the eruptive events.

**We are really grateful to Professor Dellino for his very clarifying description of the eruption phases, in the Discussion section in the new paragraph “The cause of the death and the post-mortem process” of the new version of the manuscript the description of the events has been improved following the reviewer comments.**

Regards

Pierfrancesco Dellino

Reviewer #3: There are essentially two components to your paper: the analysis of whether the plaster casts alter the chemical composition of the bone samples, as well as an interpretation on manner of death. The two topics (in my opinion) feel a bit disjointed, and having them both but in this paper seems a little forced.

**We are grateful to Reviewer #3 for her/his comments. It is important to highlight that the two mentioned topics are strictly connected to support our discussion and conclusions about the perimortem and post-mortem processes. The first important step was to develop an approach to verify if the plaster is contaminating and affecting the chemical compounds of the bones. To do this, we designed a reference set of results analysing and statistically processing the elemental contents of inhumated bones, burned bones and casts bones with casts plaster to select the cast bones minimally affected by the plaster contamination. The next step was to compare inhumated bones with Pompeii pre Vesuvian and Roma Ostiense burned bones collections, where we could observe chemical differences between these two big groups that we explain with the thermal impact in the structure of the bones that causes the loss of some major elements during post-depositional processes as confirmed by previous studies. Once we had made sure that these observations are “true” by running statistical analysis of the obtained chemical information, we could compare the cast bones with the reference set of inhumated and burned, observing that the composition of the studied casts were similar to those of the burned bones. That gives us important information to develop hypothesis about the postmortem exposure of the Pompeii remains to quite high temperature being their death caused by the asphyxia as showed by anthropological studies and the preservation of garments. Finally all supported by the volcano stratigraphic literature.**

I see nothing wrong with any of your conclusions, however they are not exactly ground breaking.

**This study shows a multidisciplinary approach cross-referencing pXRF chemical data with anthropological research and geological previous published research, supporting a death for asphyxia of the studied individuals and adding information about perimortem and post-mortem processes. Finally we developed a non-destructive methodological approach for evaluating plaster contamination to discard plaster contaminated bones and avoid misleading interpretations about bone casts original chemical contents. Therefore from a broader point of view here we present an innovative approach** **to be applied to casts from Pompeii and other Vesuvius areas shading light on the volcano eruption deaths and suggesting for the first time a protocol to be applied for the study of the casts.**

A few comments on spelling or where more clarification would be useful:

L 128 – use passive tense “only half the humeral shaft is available” rather than “we only have”

**Thanks for correcting.**

L 134 – I assume by “first half of femoral shaft” the proximal half is meant?

**Yes. Thanks this was corrected.**

L 142 – I fail to see how the sutures or teeth give indications on sex determination?

**Thanks for the correction. This is a mistake it was corrected.**

L 180 – “carpals” not carpus; metacarpal not metacarpus

**Corrected in the new version of the text**.

L 182 – humerus no humours

**Thank you for correcting.**

L 207 – one of the “previous” is redundant

**Thanks this has been corrected.**

L 331 – 334 – not sure I am following the rationale here. Also what “new authigenic phases” are referred to? A bit more information would be useful.

**Thanks, further information has been added to clarify this issue. In discussion section of the new version of the manuscript** “**The mechanisms behind these processes were further explained by Nielsen et al. [36] being bone surface apatite heavily reactive, after death the biologically formed minerals become unstable and susceptible to the burial environment, therefore Ca2+ and P4-3 ions are expose and can be totally or partially replaced by the ions of other elements.”**

L 353 – 260 – this paragraph should be introduced in the intro lit review, the discussion should onl refer to concepts previously mentioned.

**We agree with Reviewer #3, as suggested this issue was introduced with a sentence in the introduction section.**

L 386 – lixivitation is not an English word.

**Thanks for correcting. We refer at the word lixiviation as the process of lixiviating “the extraction of soluble components of a solid mixture by percolating a solvent through it”. However the word has been substituted with “leaching”.**

Reviewer #4: Overall, this is a very interesting manuscript that examines anthropological, paleopathological, and chemical analysis of human bones and casts of Pompeii victims. The paper should be published and offers new insights into the final moments of the victim's lives and their deaths. The manuscript does need substantial editing and the PDF of the manuscript has been marked up with comments, suggestions, and other edits. Some statements are overly speculative, which is noted directly on the PDF. With some editing, this paper would make a nice contribution to the literature and to PLOS ONE.

**We are really grateful for the Reviewer #4 positive response. All the comments and suggestions have been taken into accounts and reported in the new version of the manuscript revised mode with our answers as comments in the same document.**

6. PLOS authors have the option to publish the peer review history of their article ([what does this mean?](https://urldefense.com/v3/__https:/journals.plos.org/plosone/s/editorial-and-peer-review-process*loc-peer-review-history__;Iw!!D9dNQwwGXtA!UAP-2qDcHRKGLmXd9qQvIwK_V6RwxAQcIA12Msm_MgqMwOM1L47NCm5QxCXgoseR-QPMOdw8O7QAnwPUjyzu$)). If published, this will include your full peer review and any attached files.


If you choose “no”, your identity will remain anonymous but your review may still be made public.


**Do you want your identity to be public for this peer review?** For information about this choice, including consent withdrawal, please see our [Privacy Policy](https://urldefense.com/v3/__https:/www.plos.org/privacy-policy__;!!D9dNQwwGXtA!UAP-2qDcHRKGLmXd9qQvIwK_V6RwxAQcIA12Msm_MgqMwOM1L47NCm5QxCXgoseR-QPMOdw8O7QAn4xhwx6o$).

Reviewer #1: No

Reviewer #2: **Yes:** Pierfrancesco Dellino

Reviewer #3: No

Reviewer #4: No
