# Supplementary material for: The casts of Pompeii: Post-depositional methodological insights
Source: PLoS One. 2023 Aug 23;18(8):e0289378. doi: 10.1371/journal.pone.0289378 (PMC10446210; doi:10.1371/journal.pone.0289378)
Supplement: Attachment — Submitted filename: PONE__RESPONSE_TO_REVIEWERS_minors.docx [file pone.0289378.s007.docx]

PONE-D-23-12449R1

The Casts of Pompeii: post-depositional methodological insights

PLOS ONE

Dear Dr. Gallello,

Thank you for submitting your manuscript to PLOS ONE. After careful consideration, we feel that it has merit but does not fully meet PLOS ONE’s publication criteria as it currently stands. Therefore, we invite you to submit a revised version of the manuscript that addresses the points raised during the review process.


ACADEMIC EDITOR:

Dear Dr. Gallello,

We appreciate you submitting your manuscript to PLOS ONE and thank you for giving us the opportunity to consider your work.

I have completed my evaluation of your revised manuscript, which has been reviewed by two highly qualified reviewers all of whom agree it is worth to be published in PLOS ONE. Nevertheless, one of the reviewers suggested some minor changes and I also have some minor comments that should be addressed before your manuscript would be accepted.

Based on the above, I invite you to resubmit your manuscript after amending it.

PLOS ONE values your contribution and I look forward to receiving your revised manuscript.

Yours sincerely,

Dr. Olga Spekker

**AUTHORS RESPONSE:** Dear editor we are very grateful for your comments and suggestions, all the recommended changes have been amended. Furthermore the text has been double checked and revised to guarantee the quality of the manuscript and avoid as far as we can typos.

My comments:

Line 57 – “also called” instead of “also called also”

Line 66 – “the pyroclastic” instead of “that the pyroclastic”

Line 68 – “then” instead of “than”

Line 85 – “Roman” instead of “roman”

Line 89 – “In total” instead of “In resume”

Line 107 – “methods” instead of “method”

Line 116 – “was” instead of “were”

Line 122 – “demonstrated that” instead of “demonstrated”

Line 182 – “detailed description of the studied casts” instead of “the studied casts detailed description”

Line 188 – “presents hypoplasia” instead of “shows hypoplasia defects presents hypoplasia”

Line 189 – “an enthesophyte” instead of “an enthesophytes”

Lines 198–199 – “Except for Ca and P and ratios reported …” – it should be rephrased

Line 204 – “for these elements” instead of “for both these elements”

Lines 219 and 227 – “radius” instead of “radio”

Line 245 – “For, PC2” – the comma is not necessary

Line 277 – “other kind of useful” instead of “other kind useful”

Line 308 – “died of asphyxia” instead of “died for asphyxia”

**AUTHORS RESPONSE:** Thanks, the suggested changes have been made. Furthermore the word radius has been correctly written also in the Supporting Information S3.

Journal Requirements:

Please review your reference list to ensure that it is complete and correct. If you have cited papers that have been retracted, please include the rationale for doing so in the manuscript text, or remove these references and replace them with relevant current references. Any changes to the reference list should be mentioned in the rebuttal letter that accompanies your revised manuscript. If you need to cite a retracted article, indicate the article’s retracted status in the References list and also include a citation and full reference for the retraction notice.

**AUTHORS RESPONSE:** Thanks, the reference list has been revised to ensure that it is complete and correct. As far as we know retracted papers have not been cited.

[Note: HTML markup is below. Please do not edit.]

Reviewers' comments:

Reviewer's Responses to Questions

**Comments to the Author**

1. If the authors have adequately addressed your comments raised in a previous round of review and you feel that this manuscript is now acceptable for publication, you may indicate that here to bypass the “Comments to the Author” section, enter your conflict of interest statement in the “Confidential to Editor” section, and submit your "Accept" recommendation.

Reviewer #2: All comments have been addressed

Reviewer #4: All comments have been addressed

2. Is the manuscript technically sound, and do the data support the conclusions?

The manuscript must describe a technically sound piece of scientific research with data that supports the conclusions. Experiments must have been conducted rigorously, with appropriate controls, replication, and sample sizes. The conclusions must be drawn appropriately based on the data presented.

Reviewer #2: (No Response)

Reviewer #4: Yes

3. Has the statistical analysis been performed appropriately and rigorously?

Reviewer #2: (No Response)

Reviewer #4: Yes

4. Have the authors made all data underlying the findings in their manuscript fully available?

The [PLOS Data policy](http://www.plosone.org/static/policies.action#sharing) requires authors to make all data underlying the findings described in their manuscript fully available without restriction, with rare exception (please refer to the Data Availability Statement in the manuscript PDF file). The data should be provided as part of the manuscript or its supporting information, or deposited to a public repository. For example, in addition to summary statistics, the data points behind means, medians and variance measures should be available. If there are restrictions on publicly sharing data—e.g. participant privacy or use of data from a third party—those must be specified.

Reviewer #2: (No Response)

Reviewer #4: Yes

5. Is the manuscript presented in an intelligible fashion and written in standard English?

PLOS ONE does not copyedit accepted manuscripts, so the language in submitted articles must be clear, correct, and unambiguous. Any typographical or grammatical errors should be corrected at revision, so please note any specific errors here.

Reviewer #2: (No Response)

Reviewer #4: Yes

6. Review Comments to the Author

Please use the space provided to explain your answers to the questions above. You may also include additional comments for the author, including concerns about dual publication, research ethics, or publication ethics. (Please upload your review as an attachment if it exceeds 20,000 characters)

Reviewer #2: (No Response)

Reviewer #4: In this second review, I believe the author team has addressed the reviewer comments. The paper should be acceptable for PLOS ONE and will make a nice contribution to the literature on the use of archaeometric methods for studying the victims of Pompeii.

I only have a few minor edits:
Page 3, line 72: change "operated" to "conducted"
Page 3, line 85: Should roman be capitalized ("Roman")
Page 3, line 89: change "In resume" to "In total"
Page 4, line 99: "calcaneus and heel" are the same bone. Remove "and heel"
Page 4, line 104: change "were based" to "was based"
Page 4, line 117: change "materials" to "material"

**AUTHORS RESPONSE:** The Reviewer #4 minor edits have been completed as suggested. Excepting for line 104 (change "were based" to "was based) and line 117 (change "materials" to "material): disagree as plural reference and not changed.

7. PLOS authors have the option to publish the peer review history of their article ([what does this mean?](https://journals.plos.org/plosone/s/editorial-and-peer-review-process#loc-peer-review-history)). If published, this will include your full peer review and any attached files.


If you choose “no”, your identity will remain anonymous but your review may still be made public.


**Do you want your identity to be public for this peer review?** For information about this choice, including consent withdrawal, please see our [Privacy Policy](https://www.plos.org/privacy-policy).

Reviewer #2: **Yes:** pierfrancesco dellino

Reviewer #4: No

**AUTHORS RESPONSE:** We are especially grateful to the Editor and Reviewers for their work that, certainly, has consistently improved the quality of the manuscript. A sentence to thanks the Reviewers work has been added in “Acknowledgments” section.
