# Supplementary material for: The casts of Pompeii: Post-depositional methodological insights
Source: PLoS One. 2023 Aug 23;18(8):e0289378. doi: 10.1371/journal.pone.0289378 (PMC10446210; doi:10.1371/journal.pone.0289378)
Supplement: Attachment — Submitted filename: Reviewer Comments - Alapont_et_al_Manuscript.docx [file pone.0289378.s004.docx]

**The Casts of Pompeii: New insights**

Llorenç Alapont ^1^, Gianni Gallello ^1*^, Marcos Martinón-Torres ^2^, Massimo Osanna ^3^, Valeria Amoretti ^4^, Simon Chenery ^5^, Mirco Ramacciotti ^1,6^, José Luis Jiménez ^1^, Ángel Morales Rubio ^6^, M. Luisa Cervera ^6^, Agustín Pastor ^6^

^1^ Department of Prehistory, Archaeology and Ancient History, University of Valencia; Avenida de Blasco Ibáñez 28. 46010 Valencia, Spain.

^2^ McDonald Institute for Archaeological Research, University of Cambridge; Downing Street Cambridge CB2 3ER, UK.

^3^ Ministry of Cultural Heritage and Activities and Tourism; Via del Collegio Romano 27. 00186 Rome, Italy.

^4^ Archaeological Park of Pompeii; Via Villa dei Misteri, 1. 80045 Pompei, Naple, Italy.

^5^ British Geological Survey, Environmental Science Centre; Nicker Hill, Keyworth Nottingham NG12 5GG, UK.

^6^ Analytical Chemistry Department, University of Valencia; Edifici Jeroni Muñoz, Dr. Moliner 50, 46100 Burjassot, Spain

*Corresponding author. E-mail: gianni.gallello@uv.es

**Abstract**

The casts of Pompeii bear witness to the people who died during the Vesuvius 79 AD eruption. However, studies on the cause of death of these victims have not been conclusive, and the post-depositional processes and the impact of the plaster in bones have not been evaluated. Here we report on the anthropological and the first chemical data obtained from the study of six casts from Porta Nola area and one from Terme Suburbane. The comparative analyses carried out on the bone casts and other collections reveal the extent of high temperature alteration and post-depositional plaster contamination. These factors make bioarchaeological analyses difficult but still allow us to support asphyxia as the likely cause of death.

**Introduction**

The casts from Pompeii (Naples, Italy) represent one of the most important remains of the Vesuvian town; they are the expression of a dramatic event that made Pompeii an exceptional site, providing a focus of Roman culture studies [1]. Obtained in the 1870s by pouring plaster in the voids left by bodies that had decomposed under calcified volcanic ash, the casts typically contain skeletal remains embedded in a plaster cast that retains the body shape. These remains are for the most part human, giving a realistic and moving image of the people who lived and died in Pompeii, victims of the Vesuvian eruption of 79 AD. Several works have focused on identifying the cause of death of these individuals. The main contrasting hypotheses developed were either asphyxia [2,3] or body evaporation and dehydration; these last two, are based on Herculaneum population studies [4,5]. Here we report the first data from a multiproxy study carried out on seven Pompeii cast remains: six fugitives from Porta Nola area (Fig 1) and one from Terme Suburbane, cross-referencing anthropological and chemical results, thus developing a methodological approach to better define the cause of death of the studied Pompeii casts.

**Fig 1.** Location of casts from Porta Nola. (a) Original position of some of the studied Porta Nola casts (#57, #62, #58, #54, #55). (b) Map of Pompeii. (c) Detail of casts discovery area (I: Burials of Praetorians; II: Modern Masonries; III: Tomb of Obellius Firmus; IV: Porta Nola; V: Leakpan; VI: Tomb of Esquilia Polla; VII: Anonymous Tomb).

Some authors suggest that the inhabitants of Herculaneum died instantly during later pyroclastic waves due to the vaporization of body fluids, which caused the explosion of the skull and an “incomplete pugilistic attitude” from temperatures of more than 408 °C [4]. Other studies, also carried out on Herculaneum bone remains, suggested that the pyroclastic surges were below 400 °C and provoked a non-instantaneous dehydration, as indicated by the muscle contraction, "boxer position", and the preservation of collagen in the studied skeletal remains [5]. A death from asphyxia caused by ashes was also supported by different studies carried out on individuals from Pompeii [2, 3]. To confirm this last hypothesis, recently, some authors calculated that the pyroclastic density current, described as “a ground hugging gas-particle flows”, originated from the eruption column collapse or the fall of the lava dome, spread out in Pompeii at low temperature over about 17 min, which combined with the concentration of ash particles (smaller than 0.001 mm), was a long enough time to cause death by asphyxia at Pompeii. As suggested in Dellino et al. (2021) [3], the human exposure to fine ash, even at a low particle concentration, can be tolerated only for few minutes.

This research operated a bioarchaeological approach on seven Pompeii casts, followed by non-invasive chemical analysis by portable energy dispersive X-ray fluorescence spectrometer (pXRF) for the first time on these casts to determine the elemental composition in the bones and the plaster. This analytical technique has been already employed in skeletal remains to clarify taphonomic and post-depositional issues [6, 7] and in osteoarchaeological and forensic studies to identify individuals and pathologies [8, 9, 10, 11, 12]. The obtained chemical profiles of the cast bones and plaster were statistically compared with burned and calcinated bones from the Roman necropolis of Porta Nola in Pompeii, corresponding to cremated bone buried before 79 AD, as well as with cremated bones from Rome Sepolcreto Ostiense (1st-2nd Centuries AD), and buried bones from an Islamic Spanish necropolis located in Vall d’Albaida nearby Valencia (Spain).

**Methodology**

*Test samples*

Six casts (#57, #62, #58, #64, #54, #55) from Porta Nola area and one from Terme Suburbane (#34), were analysed from the anthropological and chemical (pXRF measurements) point of view. The methodological approach developed in the Casts analysis includes the pXRF measurements of cremated bones from the same area (Porta Nola) where the casts were found to control in situ contamination and post-depositional factors, those were compared with the analysis of cremated bones from another roman necropolis located in Rome. Finally, these two groups were compared with buried bones from a Spanish Islamic necropolis. In all the cases, the superficial layer of Casts bones, and the other cremated and buried bones were previously cleaned to avoid sediment contamination and plaster contamination in case of the studied casts. In resume, 20 cremated bones from Pompeii (CR. Po.) from different urns excavated in the necropolis of Porta Nola (fieldworks campaigns from 2015 to 2017) [13], 24 cremated bones (CR. Ro.) from the Necropolis of Rome Sepolcreto Ostiense (fieldwork campaign from 2018) [14] and 11 buried bones from the Islamic necropolis of Colata (Montaverner), located in the heart of the Vall d’Albaida (Valencia, Spain) (BR. Al.) [15] were sampled. See detailed information in supplementary data (Table S1).

*pXRF*

The pXRF was employed to identify the elemental composition of the analysed bones and plaster. X-ray fluorescence spectra were obtained *in-situ* using a portable S1 Titan energy dispersive X-ray fluorescence spectrometer by Bruker model (Kennewick, Washington, USA) equipped with a Rhodium X-ray tube and X-Flash® SDD detector. Geochem-trace programme was employed to quantitatively determine the elemental content of Al, Si, P, Cl, K, Ca, Ti, Mn, Cu, Zn, S, Sr and Pb. The analytical technique employed in this work were adequately validated, employing standard reference and sample control materials, in order to obtain accurate results and the instrumentation and parameters. The certified material Bone Ash NIST 1400 was used as a standard reference to evaluate the accuracy of the obtained data.

*Statistics*

Data analysis (Supplementary Data 1) was performed in R (version: 4.1.2) [16]. Principal component analysis (PCA) was employed to explore the dataset. PCA was performed in order to observe similarities among buried, cremated and cast bones, and plaster samples. All the analysed elements (Al, Si, P, Cl, K, Ca, Ti, Mn, Cu, Zn, S, Sr and Pb), and the ratios Ca/P and Sr/Ca were used as variables. Data were autoscaled prior to the PCA. Finally, plots were made for Al, Si, P, Cl, K, Ca, Ti, Mn, Cu, Zn, S, Sr and Pb concentrations and Ca/P and Sr/Ca ratios. The following R packages were used for data analysis and visualisation: factoextra (version: 1.0.7) [17] and ggplot2 (version: 3.3.5) [18].

**Results**

*Biorachaeological analysis on casts*

The Cast #57* (Fig 2) (Porta Nola. State inventory no. 90454. *new chronological classification 2019 Pompeii Archaeological Park) is an adult individual (25-30 years old) of masculine sex, divided into 6 pieces: one is the main mold (skull, chest and part of the limbs), two are from the upper left limb and three are from the lower right limb. The individual is supine and has some flexed or lopsided anatomical areas. The skull is inclined to the right, forming a 45º angle, supporting part of the occipital and parietal on the plane. The chest is straight presenting its coxofemoral joint slightly rotated towards its medial aspect (the left more than the right). Regarding the upper limbs, the right arm is incomplete at the level of the distal epiphysis of the humerus. The fragment belonging to the forearm has not yet been found. The position of the arm is stretched out at an angle of 90 degrees to the chest of the individual. From the left arm we only have half of the shaft of the humerus, which is attached to the chest. The left leg has an antero-lateral appearance, that is, the leg is slightly rotated inward and possibly the foreleg bent backwards giving an angle greater than 90º. It should be noted that this is fractured at the knee. On the back. The remains of the proximal epiphysis of the broken tibia have been located as a result of the insertion of the irons from old restorations. Finally, the right leg has a slightly flexed position showing its antero-lateral face, with an angle of more than 90º, and limb is fragmented at the height of the first half of the femur shaft. Also, a loss of plaster was observed on its back and front.

**Fig 2.** Cast #57 and pXRF measuring points (1-6).

The Cast #62* (Fig. 3) (Porta Nola. State inventory no. 90453. *new chronological classification 2019 Pompeii Archaeological Park) is an adult individual (25-30 years old) of female sex (macroscopic observation of the cranial bones and their sutures and of the front teeth). She has interlaced into the left hand-forearm, a rope that would go over the left shoulder to carry a sack or bag with belongings. The original position of the subject was prone (face-down) with the head resting on the face, slightly tilted to the right resting on the facial bones, mandible, frontal and left zygomatic. The right arm was together with the elbow, fully flexed and bent with the hand under the right shoulder. The left arm was with the elbow flexed to 30º, with the hand under the left hemithorax. The legs were parallel following the longitudinal axis of the body. The right leg with the knee extended and the left with the knee slightly bent.

**Fig 3.** Cast #62 and pXRF measuring points (1-6).

The Cast #58* (Fig 4) (Porta Nola. State inventory no. 90455. *new chronological classification 2019 Pompeii Archaeological Park) is an adult individual (20-25 years old) of masculine sex. This individual was originally found supine, with the upper extremities separated from the body. It was divided into seven parts before the restoration and the following bones can be observed: frontal, parietal and left temporal; part of the left molar, zygomatic, the mandible is visible from the lower part of the left branch at the height of the mental hole, part of the teeth of the upper jaw and mandible are also visible. It appears to preserve the morphology of the right ear. It has stone concretions (lapilli) in the facial area (maxillary and nasal), more prominent in the right than left area. There are seven ribs on the left side (4th to 10th). As for the upper limbs, the bones of the right humerus are visible (which has been moved by inserting towards the thorax with a pause at the level of the Coronoid Fossa).

**Fig 4.** Cast #58 and pXRF measuring points (1-6).

Head facing forward but slightly inclined to the left, the trunk is straight, the right arm is apparently attached to the torso and bent from the elbow, leaving the forearm separated from the body, forming an angle of 65 ° to the arm. The left arm is raised to shoulder height, bent from the elbow towards the individual's face, forming an angle of approximately 90°. It appears in a sitting position, with its left leg bent at the knee at a 90°angle. The right leg is semi-flexed and turned clockwise, leaving the medial part exposed. Fragments associated with the individual:

The right forearm from the elbow joint to the proximal phalanges; the section of the epiphysis of the distal humours, as well as the articular facets of the proximal distal phalanges can be seen; a fragment of plaster that match only where the plaster tail joint, which is only where the elbow joint, together with the fragment described above; the left arm, from the first third of the distal humerus shaft and epiphysis up to the distal epiphysis of the metacarpus and the fragment of one of the proximal epiphyses of the proximal phalanges can be seen.

Some carpus (such as the navicular and lunate), the proximal epiphysis of the metacarpus, the proximal epiphysis of the next second phalanx fragment are also visible. There is a fracture of the area beyond the shaft of the humours, which appears dissected and continues in the mould of the individual. In the same skeletal section, there is a fragment of a rib and the anterior is part of the distal epiphysis of the radius, and in its side the flexed elbow joint (humerus, ulna and radius) is perfectly visible. These three bones are visible in the following way: Cube from the extreme proximal epiphysis up to half of the diaphysis; the radius from the end of the proximal epiphysis to the first third of the diaphysis, and the distal epiphysis of the left humerus.

The leg from the proximal epiphysis / extremity of tibia and fibula to the heel, calcaneus bone, cuboid and third and second cuneiform with the left lower limb of the mould are still visible. Parallel to the fibula runs a root that has left marks on the bone, of a reddish hue. In this area there are stone concretions that involve practically all the legs.

The right foot has preserved its morphology in the mould; part of the trochlea of the talus and part of the bone of the metacarpus and proximal phalanges.

The Cast #64* (Fig 5) (Porta Nola. State inventory no. 90461. *new chronological classification 2019 Pompeii Archaeological Park). It was identified as an adult individual between 45 and 50 years old, probably male, very altered and remodelled in an old restoration, especially in the zygomatic-facial area. The ends of both legs are missing from the knee joint. In the right knee, a chronic and prolonged arthritic lesion stands out, a fact that indicates hard physical activity. Individual originally arranged supine, with the upper extremities separated from the body. The right arm with the elbow is slightly flexed and the left with the elbow joint flexed 90º.

**Fig 5.** Cast #64 and pXRF measuring points (1-6).

The Cast #54* (Fig 6) (Porta Nola. State inventory no. 90452. *new chronological classification 2019 Pompeii Archaeological Park) it is an individual exhibited at the Pompeii amphitheatre exhibition, therefore it has not been possible to carry out the full investigation (3D scan, photogrammetry) and the anthropological and paleopathological studies. Using the data from our previous 2012 previous research, it could be estimated that it is a female adult (35-40 years) determined by the macroscopic observation of the cranial, parietal and occipital bones and lambdoid and sagittal sutures. The individual was found on the left lateral decubitus, with the head resting on the left temporal parietal and the right arm with the elbow fully flexed and the forearm next to the chest. The left arm flexed 90º with the forearm next to the abdomen is in a position to hold a cane, branch, or crook. The right leg is with the knee bent 90º on the left leg while the left leg is slightly bent.

**Fig 6.** Cast #54 and pXRF measuring points (1-6).

The Cast #55* (Fig 7) (Porta Nola. State inventory no. 90462. *new chronological classification 2019 Pompeii Archaeological Park) it was identified as an adult individual between 25 and 30 years old, male, on the right arm showing a perimortem fracture of the distal third of the shaft of the humerus where a thick tree branch appears. This Individual originally arranged supine, with the upper extremities separated from the body, with the forearms and hands on the head. Both arms appear symmetrical and parallel with the elbows flexed 30º. The legs are separated, symmetrical and parallel, following the longitudinal axis of the body with both knees bent 45º.

**Fig 7.** Cast #55 and pXRF measuring points (1-6).

The Cast #34* (Fig 8) (Terme Suburbane. State inventory no. 90459. *new chronological classification 2019 Pompeii Archaeological Park) is an adult individual (20-25 years) probable male, identified by dimensions of the right femur. The original position of the subject was right lateral decubitus, with the head resting on the right zygomatic, parietal and temporal bones. The right arm was with the elbow flexed, separated from the body. The legs were parallel following the longitudinal axis of the body, both legs with the knees bent at 90º.

**Fig 8.** Cast #34 and pXRF measuring points (1-6).

Furthermore, the paleopathological data showed interesting features in some of the studied individuals. The Cast #57 shows dental wear of the incisors and canines of the upper jaw, product of its axial shaft, and erosion of the lower incisors. Individual #58 also presents hypoplasia in the left lateral incisor and dental wear on the lower left lateral incisor, and also an enthesophytes of the left patella while Cast #64 presents osteoarthritis in the right knee end and in the medial condyle of left femur. The radiological examination also revealed traumatic perimortem fractures in the tibia and fibula of the right leg of the cast #58 and in the right humerus of the Cast #55. These fractures appear to have occurred close to the time of death of the individuals. No obvious pathologies in the individuals #62 and #54 from Porta Nola and #34 from Terme Suburbane, were observed.

*pXRF analysis*

Multielement analysis results (see the measuring points in Figs 2-8) are shown in the supplementary data (Table S1) and can be visualized in the Supporting Information (Supplementary Figs).

The plots suggest that both cast and plaster samples have elemental levels similar to bones for Pb (Supplementary Fig S1), Mn (Supplementary Fig S2) and Al (Supplementary Fig S3). The different groups of samples also show similar amounts of Zn (Supplementary Fig 4), although some casts are characterized by higher amounts of these elements, especially in the analyzed teeth of Cast #57 and Cast #62 (TT). On the other hand, bones are richer in Ca (Fig 9) and P (Fig 10) than plaster samples, and buried bones (BR. Albaida) show the highest mean concentrations for both these elements. Several cast measured points fall within a standard deviation of the two cremated bones groups and it should be pointed out that Cast #34 has the lowest concentrations for both elements (Ca and P). Most of the plaster samples show high concentrations of K (Supplementary Fig 5), Cl (Fig S6), Ti (Supplementary Fig S7) and Si (Supplementary Fig S8), and several casts show higher concentrations than bones, although cremated bones from Rome are characterized by higher elemental levels than those from Pompeii and of the inhumated ones. Plaster samples have the highest amounts of Fe (Supplementary Fig S9), Sr (Supplementary Fig S10) and S (Supplementary Fig S11). Casts are characterized by levels of Fe similar to those of cremated bones, while, in most of the cases, they have levels of Sr and S higher than the former ones. Concerning the calculated ratios, plasters are enriched in Ca over P (Fig 11) and in Sr over Ca (Fig 12), which is reflected also in some of the studied casts, such as Cast #34.

**Fig 9.** Ca concentrations for all the analyzed materials**.** Cast bones and plaster (P57, P62, P58, P34, P64, P54, P55), cremated bones from Pompeii (CR. Po.) and Rome (CR. Ro.), and buried bones from the Islamic necropolis of Vall d’ Albaida (BR. Al.). In the graph, for each cast the kind of bone is indicated: cranium (CR), parietal (PR), occipital (OC), frontal (FR), maxilla (MX), mandibular (MD), tooth (TT), radio (RD), peroneal bone (PR), tibia (TB), femur (FM). •: mean; ♦: one standard deviation.

**Fig 10.** P concentrations for all the analyzed materials. Cast bones and plaster (P57, P62, P58, P34, P64, P54, P55), cremated bones from Pompeii (CR. Po.) and Rome (CR. Ro.) and buried bones from the Islamic necropolis of Vall d’Albaida (BR. Al.). In the graph for each cast the kind of bone is indicated: cranium (CR), parietal (PR), occipital (OC), frontal (FR), maxilla (MX), mandibular (MD), tooth (TT), radio (RD), peroneal bone (PR), tibia (TB), femur (FM). •: mean; ♦: one standard deviation.

**Fig 11.** Ca/P ratio for the analyzed materials. (a) Diagram for all the analyzed materials. (b) Diagram excluding plaster (in the range up to 20). Cast bones and plaster (P57, P62, P58, P34, P64, P54, P55), cremated bones from Pompeii (CR. Po.) and Rome (CR. Ro.) and buried bones from the Islamic necropolis of Vall d’Albaida (BR. Al.). In the graph for each cast (Cast) the kind of bone is indicated: cranium (CR), parietal (PR), occipital (OC), frontal (FR), maxilla (MX), mandibular (MD), tooth (TT), radio (RD), peroneal bone (PR), tibia (TB), femur (FM). •: mean; ♦: one standard deviation. Orange line shows the interval ratio (2.06-2.45) in fresh bones.

**Fig 12.** Sr/Ca ratios for all the analyzed materials. Cast bones and plaster (P57, P62, P58, P34, P64, P54, P55), cremated bones from Pompeii (CR. Po.) and Rome (CR. Ro.) and buried bones from the Islamic necropolis of Vall d’Albaida (BR. Al.). In the graph for each cast the kind of bone is indicated: cranium (CR), parietal (PR), occipital (OC), frontal (FR), maxilla (MX), mandibular (MD), tooth (TT), radio (RD), peroneal bone (PR), tibia (TB), femur (FM). •: mean; ♦: one standard deviation.

*Principal component analysis (PCA)*

The principal component analysis (PCA) of the chemical data was carried out to observe variation of cast bones with respect to the comparative sets of burned bones, buried bones and plasters (Fig 13). The first two PCs explain 63.7% of the overall variance in the dataset.

As can be observed in the samples/scores plot (Fig 13a), plaster samples have higher PC1 scores than bone samples. It is worth noticing that samples of buried bone group together in the third quadrant of the diagram, while those from cremated bones are scattered on both PC1 and PC2. Most of the cast samples fall within cremated bones. The colored triangles referring to cremated bone colors do not show a clear grouping although the majority of the brownish bones are similar to the buried ones, which is consistent with their exposure to lower temperatures. Concerning cast samples, the majority of the cast bones are located close to cremated bones, although some fall between bones and plaster (C34.1, C34.4, C55.5, C57.4) or close to plaster (C34.3), probably due to the results of being encapsulated by plaster (Fig 13a). Variables/loadings plots (Fig 13b) indicate that scores for PC1 are mainly driven by P and Ca concentrations in the negative direction and by most of the other elements and Ca/P and Sr/Ca ratios in the positive one. For, PC2, S, Sr and ratios are the most influential variables in the negative direction, and Al, Si, Ti, Mn, Zn and Pb in the positive one. The second PC (13.6%) provides little information to differentiate between the classes (Fig 13a).

**Fig 13.** Principal component analysis of chemical data from Porta Nola casts and test samples. PCA scores plot with the labeled casts measured points. (a) Samples/scores plot for cast (Cast), plaster (Plaster) and cremated bones from Pompeii (CR. Pompeii) and Rome (CR. Rome) and buried bones from the Islamic necropolis of Vall d’ Albaida (BR. Albaida). Colored cremated sample points refer to bone color. (b) Variables/loadings plots for PC1 and PC2.

Overall, the obtained results show that phosphorous and calcium concentrations are the most important variables in the negative direction of PC1, being particularly high in buried bones. The role of calcium and phosphorus concentrations can be appreciated in the PCA (Fig. 13b). The individual #34 suffered major plaster contamination in tibiae (C34.1 and C34.3) and femur (C34.4) as confirmed by the calcium, phosphorous and calcium/phosphorous ratio graphs (Figs 9, 10, 11), while the parietal of the same individual (C34.5) seems to suffer only minor plaster contamination. Ca/P ratio, calculated from Hancock et al. [19] for fresh bones values, was settled between 2.17-2.31, values close to the bone biological range will be indicative of the concurrence of both dissolution and recrystallization processes, values above this range will be indicative of processes of precipitation of new authigenic phases (Fig 10) [20]. The three analysed cast teeth; C57.5, C62.2 and C55.3 and the Pompeii burned tooth T055 show a Ca/P ratio in the interval of fresh bones suggesting an insignificant plaster contamination (Fig. 11). More interesting, in PCA (Fig 13) can be observed that the three teeth have a chemical profile similar to the burned bones including the tooth T055 indicating a proportional decrease of Ca and P in these samples. No clear differences were observed among casts, plaster, cremated and buried bones (see also the Supplementary Figs S1 to S11) for other elements.

Thus, the use of plaster as a consolidant significantly affected the chemistry of some analysed cast bones, while at the same time it helped preserve other useful information. There are many examples of garments and objects such as a tied sack or objects made by leather or hemp, preserved during the burial by ashes, which were identified in the casts of Porta Nola through the plaster imprint. Similarly, textile impressions have been observed in several of the studied casts (casts #56, #57, #34, #55 and #58), providing opportunities for further research. In some cases, fine fabrics were observable (cast #57, Fig. 14), while in other cases different textiles, most likely made of wool, were identified (casts #34 and #56). In addition, the casts preserve a body posture (cast number #34 and #62) indicating that the individuals were covering themselves up in an attempt to provide protection against the ash fall.

**Fig 14.** Clothing details of the Cast #57.

**Discussion**

A key question of this study is how our multiproxy analysis can provide data to test hypotheses about the mode of death of Porta Nola fugitives. Some of the Pompeii bodies were found under more than three meters of lapilli, buried under the building collapses and blocked by pumice, while those who were outside, ended their existence under the collapse of the houses, devastated by the rocks and pyroclastic material expelled by the volcano. This likely happened during the first few hours of eruption as suggested by the stratigraphic studies of Luongo and colleagues [21]. Nevertheless, the stratigraphic position of the studied Porta Nola fugitives indicate that they survived the early catastrophic event and died 20 hours later from the start of the eruption after the pyroclastic deposition of lapilli [22].

Focusing on the victims of Porta Nola, the stratigraphic data [23,21] suggest that these people were fleeing the city when the rain of lapilli ended. Walking on the layer of pumice would have been quite difficult and some of them employed branches as a walking stick (see Cast #54); the suspended ashes would have reduced visibility and the finer ashes entered the eyes, nose, and mouth. There are many studies on the devastating effects of volcanic ash and gases in the body; when gasses are inhaled and the ash enters in the respiratory system, breathing becomes impossible [24]. These problems would have been compounded by collapsed buildings and the fallen trees as evidenced by various casts of tree trunks in the Porta Nola area. For example, the aforementioned Cast #55 corresponds to a man lying on his back with a large tree branch fallen on his right arm, which caused the humerus perimortem fracture.

The victims of Porta Nola do not show a "pugilistic" position and they are not carrying out any action or movement despite their attempts to escape. All of them appear to be lying on their back or prone or on their side, in a relaxed position; some of them covering themselves with garments. This position suggests that ashes and volcanic gases caused the death of the exhausted and asphyxiated fugitives of Porta Nola, before being hit by the pyroclastic wave.

Nonetheless, we should take into account that the extreme heat from the pyroclastic flows, which covered the Porta Nola victims in ash and mud, could have had an impact on the bodies (perimortem or postmortem), without being related to the cause of death. The temperature could be very significant data if cross-referenced with ash stratigraphy and taphonomy. Our results show that the analysed cast bones negligibly affected by plaster contamination (see the aforementioned Ca/P ratio in teeth) have a similar chemical profile to cremated individuals from necropolises in Pompeii (pre-eruption necropolis) and in Rome, and different from buried bones (medieval necropolis). The higher levels of calcium and phosphorus in buried bones compared to cremated ones can been explained by the thermal impact suffered by bones, which induces both a chemical and mineralogical change in the bones with the consequence of a major leaching of these two elements. Probably the thermal impact together with post-depositional processes induced the lixiviation of carbonates and phosphates and this could explain the lower calcium and phosphorous concentrations in burned bones [25, 26]. All this especially clarified the fact that, if the bones in Porta Nola were impacted by a high temperature, this would have happened in a post-depositional condition, this is also confirmed by the presence of textiles fingerprinted by the plaster (Fig 14). The process can be described following the stratigraphy of the deposits of Sigurdsson et al. (1982) [22]: first, the individuals suffered asphyxia and were killed by fine ashes while they were laying on the ground trying to cover themselves with clothes, and with the fine ashes taking the shape of surrounding objects, including fine textiles. Later, the dead bodies were covered by the ground surge materials. Finally, the bodies under the high temperature (over 250 degrees) of the pyroclastic flow, suffered an “oven effect” and the “cooked” ashes left their fingerprint in a cavity, with just bones being left for many centuries, potentially suffering post-depositional processes before their discovery and infilling with plaster by archaeologists.

**Conclusion**

For the first time the bone remains of fugitives from the Pompeii eruption have been directly analysed, employing non-destructive analytical techniques (pXRF). Elemental profiles were determined providing important data that cross-referenced with anthropological, paleopatological, taphonomic and stratigraphic results are clearly helpful in the reconstruction of the perimortem and postmortem events concerning the history of these individuals.

Our developed hypothesis for the cause of death is only applicable to the context studied. It is likely that the catastrophic eruption killed people in different ways and all the scenarios discussed should be taken into account. Generalizing and supporting a sole hypothesis of death becomes over reductive.

Moreover, the use of chemical analyses on the cast skeletons is encouraged to assess the extent of post-depositional alteration and plaster contamination prior to any biomineral or bimolecular analyses, in order to avoid misleading interpretations.

This proposed approach may shed light on resolving probable causes related to the deaths during the volcano eruption and create the prerequisites for a protocol to be applied at casts from Pompeii and other Vesuvian areas.

**Data and materials availability**

All data are available in the main text or in the Supplementary Information.

**Acknowledgments**

The authors would like to thanks all the collaborators and students for their support during the 2018 Pompeii Archaeological Park fieldwork, and also all the public institutions that have contributed to the realization of this study. G. G. acknowledges the Spanish Ministry of Science and Innovation and Ministry of Universities for the grant BEAGAL18/00110, and the European Commission for funding the project H2020-MSCA-IF-2015-704709-MATRIX. M.R., M.L.C., A.P. and A.M.R. acknowledge the Ministry of Education, Research, Culture and Sport of the Valencian Generality for funding the project PROMETEO 2019-056.

**Author contributions**

L.A. and G.G: Conceptualisation, Methodology, Investigation, Formal Analysis, Supervision, Project Administration, Visualisation, Writing – Original Draft Preparation; Writing – Review & Editing. M.M., S.C., M.R., J.L.J., A.M.R., M.L.C. and A.P.: Investigation, Validation, Visualisation, Writing – Review & Editing . M.O. and V.A. Investigation, Validation, Writing – Review & Editing.

**References**

1. Osanna M, Capurso A, Masseroli SM (editors). I Calchi di Pompei da Giuseppe Fiorelli a Oggi. Pompeii: Archaeological Park of Pompeii; 2021.

2. Dellino P, Dioguardi F, Isaia R, Sulpizio R, Mele, D. The impact of pyroclastic density currents duration on humans: the case of the AD 79 eruption of Vesuvius. Sci. Rep. 2021; **11:** 4959.

3. Giacomelli L, Perrotta A, Scandone R, Scarpati C. The eruption of Vesuvius of 79 AD and its impact on human environment in Pompeii. Episodes 2003; 26(3): 235-238.

4. Petrone P, Pucci P, Vergara A, Amoresano A, Birolo L, Pane F, et al. A hypothesis of sudden body fluid vaporization in the 79 AD victims of Vesuvius. PLOS ONE 2018; 13(9): e0203210.

5. Martyn R, Craig OE, Ellingham STD, Meez I, Fattore L, Sperduti A, et al. Re-evaluation of manner of death at Roman Herculaneum following the AD 79 eruption of Vesuvius. Antiq 2020; 94(373): 76-91.

6. Pessanha S, Carvalho M, Carvalho ML, Dias A. Quantitative analysis of human remains from 18th–19th centuries using X-ray fluorescence techniques: The mysterious high content of mercury in hair. J Trace Elem Med Biol 2016; 33: 26-30.

7. Janiga M, Janiga M. Different accumulation of some elements in the fry and adults of alpine bullheads (Cottus poecilopus). Environ Sci Pollut Res (2023); 30: 44724-44732.

8. Gonzalez-Rodriguez J, Fowler G. A study on the discrimination of human skeletons using X-ray fluorescence and chemometric tools in chemical anthropology. Forensic Sci. Int. 2013; 231: 407.e1–407.e6.

9. Perrone A, Finlayson JE, Bartelink EJ, Dalton KD. Application of portable X-ray fluorescence (XRF) for sorting commingled human remains. In: Adams BJ, Byrd JE (editors). Commingled Human Remains. San Diego: Academic Press; 2014. pp. 145-165.

10. Finlayson JE, Bartelink EJ, Perrone A, Dalton K. Multimethod resolution of a small-scale case of commingling. J Forensic Sci 2017; 62: 493-497.

11. Gomes RAMP, Catarino L, Santos AL. Anemia, cribra cranii and elemental composition using portable X-ray fluorescence: A study in individuals from the Coimbra Identified Osteological Collections. J Archaeol Sci 2021: 136: 105514.

12. Kilburn NN, Gowland RL, Halldórsdóttir HH, Williams R, Thompson TJU. Assessing pathological conditions in archaeological bone using portable X-ray fluorescence (pXRF). J Archaeol Sci 2021; 37: 102980.

13. Diez Castillo A, Gómez Puche M. Excavaciones arqueológicas en el yacimiento de Colata (Montaverner, Valencia). In: Terol i Reig V, Casanova E (editors). II Congrés d’Estudis de la Vall d’Albaida (Ontinyent, 5-7 de novembre de 2004): actes. Valencia: Institució Alfons el Magnànim; 2008. pp. 473-495.

14. Kay S, Ceccarelli L, Alapont L, Albiach R. Excavations at the Necropolis of Porta Nola. New evidence for production and circulation in Pompeii. In: Osanna M, Toniolo L. (editors). Fecisti Cretaria. Dal Frammento Al Contesto: Studi Sul Vasellame Ceramico del Territorio Vesuviano. Roma - Bristol: «L’Erma» di Bretschneider; 2020. pp. 340-342.

15. Marcelli M, Cicone C, Alapont Martín Ll, Sastre Morro M, Evans SF, Lhériteau M. Nuove ricerche sui colombari del Sepolcreto della via Ostiense. Analisi dei resti antropologici e archeologici (Mun. VIII). Bull Commissione Archeologica Comunale Roma 2021, 121: 289-307.

16. R Core Team. R: A language and environment for statistical computing. Wien: R Foundation for Statistical Computing; 2021.Available: https://www.R-project.org/

17. Kassambara A, Mundt F. factoextra: Extract and Visualize the Results of Multivariate Data Analyses. R package version 1.0.7; 2020. Available: https://CRAN.R-project.org/package=factoextra

18. Wickham H. ggplot2: Elegant Graphics for Data Analysis. New York: Springer-Verlag; 2016.

19. Hancock RGV, Grynpas MD, Åkesson K, Obrant JT, Kessler MJ. Baselines and variabilities for major and trace elements in bone. In: Lambert JB, Grupe G (editors). Prehistoric Human Bone: Archaeology at the molecular level. Heidelberg: Springer Berlin; 1993. pp. 189-201.

20. Nielsen-Marsh CM, Hedges REM. Dissolution experiments on modern and diagenetically altered bone and their effect on the infrared splitting factor. Bull Soc Géol France 1997; 168: 485-490.

21. Luongo G, Perrotta A., Scarpati C., De Carolis E., Patricelli G., Ciarallo A. Impact of the AD 79 eruption on Pompeii, II. Causes of death of the inhabitants inferred by stratigraphic analysis and areal distribution of the human causalities. J Volcanol Geotherm Res 2003; 126: 169-200.

22. Sigurdsson H, Cashdollar S, Sparks SRJ. The Eruption of Vesuvius in A. D. 79: Reconstruction from Historical and Volcanological Evidence Source. Am J Archaeol 1982; 86: 39-51.

23. Doronzo DM, Di Vito MA, Arienzo I, Bini M, Calusi B, Cerminara M, et al. The 79 CE eruption of Vesuvius: A lesson from the past and the need of a multidisciplinary approach for developments in volcanology. Earth Sci Rev 2022; 231: 104072. doi: 10.1016/j.earscirev.2022.104072

24. Zanella E, Gurioli L, Pareschi MT, Lanza R. Influences of urban fabric on pyroclastic density currents at Pompeii (Italy): 2. Temperature of the deposits and hazard implications. J Geophys Res Solid Earth 2007; 112: 1-18.

25. Gallello G, Kuligowski J, Pastor A, Diez A, Bernabeu J. Biological mineral content in Iberian skeletal cremains for control of diagenetic factors employing multivariate statistics. J Archaeol Sci 2013; 40: 2477–2484.

26. Snoeck C, Lee-Thorp JA, Schulting RJ. From bone to ash: Compositional and structural changes in burned modern and archaeological bone. Palaeogeogr Palaeoclimatol Palaeoecol 2014; 416: 55-68.
